# Combined Effect of Lead Exposure and Allostatic Load on Cardiovascular Disease Mortality—A Preliminary Study

**DOI:** 10.3390/ijerph18136879

**Published:** 2021-06-26

**Authors:** Emmanuel Obeng-Gyasi, Alesia C. Ferguson, Katherine A. Stamatakis, Michael A. Province

**Affiliations:** 1 Department of Built Environment, North Carolina A&T State University, Greensboro, NC 27411, USA; acferguson@ncat.edu; 2 Environmental Health and Disease Laboratory, North Carolina A&T State University, Greensboro, NC 27411, USA; 3 Department of Epidemiology and Biostatistics, College for Public Health & Social Justice, Saint Louis University, St. Louis, MO 63103, USA; katie.stamatakis@slu.edu; 4 Department of Genetics, Washington University School of Medicine St. Louis, St. Louis, MO 63130, USA; mprovince@wustl.edu

**Keywords:** stress, mixed exposures, allostatic load, cardiovascular disease mortality, lead exposure

## Abstract

This study explores the combined effect of lead (Pb) exposure and an index of chronic physiological stress on cardiovascular disease mortality using data from the National Health and Nutrition Examination Survey (NHANES) 1999–2008 linked to 1999–2014 National Death Index data. Chronic physiological stress was measured using the allostatic load (AL) index, which was formed by analyzing markers from the cardiovascular, inflammatory, and metabolic systems, with Pb levels, assessed using blood lead levels (BLL). The dataset was analyzed with statistical techniques to explore (a) the relationship between Pb exposure and AL, and (b) the combined role of Pb and AL on cardiovascular disease mortality. Results indicated that AL was more elevated in those with BLLs above the 50th percentile in the US population and that those with elevated AL were more likely to have high BLL. Finally, the interaction of AL and BLL significantly increased the likelihood of cardiovascular disease mortality. These findings highlight the need for considering the totality of exposures experienced by populations to build holistic programs to prevent Pb exposure and reduce stressors to promote optimal health outcomes and reduce cardiovascular mortality risk.

## 1. Introduction

In the US population, cardiovascular diseases are the leading cause of mortality [1]. Numerous epidemiological studies have linked lead (Pb) exposure with adverse cardiovascular disease outcomes [2,3,4,5]. According to the Centers for Disease Control and Prevention (CDC), any exposure to Pb can induce disease and dysfunction in children [6]. 

Lead (Pb) is an environmentally and biologically persistent source of adverse outcomes affecting nearly all the organ systems in the human body [7,8,9,10,11,12]. Sources of Pb exposure in the United States include Pb-based paint, contaminated soil, drinking water, workplace sources (e.g., battery manufacturing), and Pb-contaminated imported goods [13]. Adults are primarily exposed to Pb in the workplace [14] with 5 μg/dL used as the case classification by the Adult Blood Lead Epidemiology and Surveillance (ABLES) to indicate an elevated BLL for surveillance purposes [15]. However, the US Centers for Disease Control and Prevention (CDC) have concluded that no Pb exposure level is safe for adults as adverse health outcomes have been documented at all exposure concentrations [6,16]. Race is a critical factor in Pb exposure risk in the United States, with environmental inequities leaving Black populations disproportionately exposed. Owing in part to environmental racism, social and environmental conditions such as residential location, education, and employment have historically led to greater exposure in minority populations to environmental hazards such as Pb, with prevention and remediation of exposure sources being slow for affected groups [17,18]. 

Cardiovascular disease risk factors, such as hypertension, diabetes mellitus, and cardiovascular diseases, such as heart failure and ischemic heart disease, play a significant role in cardiovascular disease mortality [19]. Environmental contaminants such as Pb may also play a role in cardiovascular disease mortality. The role of Pb in cardiovascular disease mortality was explored in a 2018 study by Lanphear and colleagues, in which they estimated that 256,000 premature deaths from cardiovascular diseases, including 185,000 deaths from ischemic heart disease, may be linked to Pb exposure adults [20]. 

Allostatic Load (AL), a measure of chronic stress, represents the hypothalamic–pituitary–adrenal (HPA) axis in an over-activated state and involves dysregulation of multiple physiological systems [21]. Dysregulation of the HPA axis, critical for regulating neuroendocrine system responses to stressful stimuli, has been associated with cardiovascular dysfunction [22]. AL offers vital insight into the effects of chronic stress on health outcomes [23,24,25,26]. 

Epidemiological studies have demonstrated that chronic stress predicts the occurrence of coronary heart disease (CHD), with even short-term emotional stress triggering cardiac events [27,28]. Studies have also found that stress plays a role in cardiovascular mortality [29,30]. 

Pb may also increase the stress response. In a study of Pb-exposed workers, Chang and colleagues found high plasma norepinephrine but normal plasma dopamine and epinephrine levels, pointing to heightened sympathetic nervous system activity [31]. Gump and colleagues found that after an acute stressor, increasing prenatal and postnatal blood Pb levels were independently associated with significantly heightened salivary cortisol responses [32], with Pb’s effect on salivary cortisol confirmed by others [33]. Regarding chronic stress, Zota and colleagues found that AL may amplify the adverse effects of Pb on blood pressure [33], with other studies confirming Pb exposure at lower levels contributing to AL and in adults [34].

People’s daily activities, including their diet, neighborhood, environmental exposures, social interactions, and lifestyle behaviors such as alcohol and smoking, affect health outcomes. The exposome, a concept that seeks to assess the cumulative measure of environmental impacts and related biological responses throughout the life-course, including exposures from diet, behavior, the environment, and endogenous processes, better reflects populations’ exposure risk. The combined effect of Pb and life stressors on cardiovascular disease mortality is thus critical to study since both may have a cumulative impact on the cardiovascular system.

Prior studies by the lead author have demonstrated potential associations between AL and cardiovascular dysfunction among those exposed to differing concentrations of Pb, but more information is needed to understand how the combination of Pb and AL contributes to cardiovascular disease mortality [35].

This study had two objectives:

1: Determine the association between Pb and AL among adults.

2: Determine the association between the combined effect of AL and Pb on cardiovascular disease mortality among adults.

## 2. Materials and Methods

This study examined the association between BLL and AL, and the combined effect of BLL and AL on cardiovascular disease mortality in adults using data from the National Health and Nutrition Examination Survey linked to the National Death Index. Biological and clinical markers from multiple physiological systems such as the cardiovascular, inflammatory, and metabolic systems were used to develop the AL index. AL, an established index of chronic stress [21,36,37,38,39,40,41], is a suitable means to explore the role of Pb and chronic stress in adults, as it represents the cumulative wear-and-tear on the body. 

### 2.1. Measures

Allostatic Load Index:

Informed by prior studies [35,42,43], a cumulative index of physiologic dysfunction of the cardiovascular (SBP, DBP, triglycerides, HDL cholesterol, total cholesterol), inflammatory (CRP), and metabolic systems (BMI, hemoglobin A1C, albumin, creatinine clearance) was developed. AL markers were divided into quartiles based on their distribution within the database. High-risk for each biomarker was considered to be the top 25% in the distribution for all markers apart from albumin, creatinine clearance, and HDL cholesterol, for which the bottom 25% of the distribution was considered to have the highest [44,45,46,47,48,49,50]. Each individual in the study was assigned a value of 1 if they are in the high-risk category or a 0 if in the low-risk category for all markers to calculate a total AL value out of 10. Clinical and laboratory collection and analysis of markers and variables of interest have been described elsewhere [35,43]. 

### 2.2. Data and Study Design

The relationships in this study were explored using NHANES 1999 to 2008. Mortality was determined from linked 1999–2014 National Death Index data. NHANES data is a stratified, multistage probability sample of civilian non-institutionalized individuals in all of the 50 US states, including the District of Columbia. Technical details of the survey, including sampling design, data collection protocols, and data availability, are freely available on their website. Data collection for NHANES makers of interest have been described elsewhere [35]. This study used de-identified secondary data; hence, the study did not require IRB approval. 

### 2.3. Data Analysis

Variables of interest were explored in adults aged 20 or older in those with BLL above the 50th percentile in the US population as compared to those with BLL below the 50th percentile. This study included an overall eligible sample of 28,852 adults with 52.05% being female.

Linear regression and logistic regression were used to explore the association between AL and BLL in adults by dichotomizing low AL ( <3 or <4 ) vs. high AL ( ≥3 or ≥4) for logistic regression, as high AL subjects using this definition have been consistently shown in the literature to be at high risk of adverse health outcomes from chronic stress [51,52,53].

Synergistic effects of co-exposures to high values of both blood Pb and AL on cardiovascular mortality were determined by estimating odds ratios concerning exposures to low values of both blood Pb and AL.

Cox proportional hazards regression was used to model the risk of death with less-stressed (AL <3) and low Pb exposure (BLL < 50th percentile in population) participants as the reference groups. Additional covariates used were gender, BMI, alcohol consumption, smoking, and country of birth, as these factors were different across Pb exposure levels and the literature has demonstrated they may confound variables of interest. 

Stata SE/16.0 (StataCorp, College Station, TX, USA) was used for the analysis as it factored in the complex design to ensure the analysis reflected the proper weights.

## 3. Results

### 3.1. Summary Statistics of Variables of Interest

Variables of interest we explored above and below the median BLL (1.55 μg/dL) were among adults aged 20–85 years old (Table 1). The mean AL above the 50th percentile BLL was significantly more elevated than below the 50th percentile, indicating the role Pb may play in elevating AL.

The proportion of non-Hispanic Blacks and Mexican Americans increased above the 50th percentile in BLL as compared to other ethnicities while the proportion of non-Hispanic Whites significantly (*p* < 0.05) decreased. 

### 3.2. Association between AL and BLL

Linear and logistic regression models indicated that blood Pb was significantly associated with AL (Table 2). For logistic regression models, the threshold of an AL of 3 and 4 were both significantly associated with BLL even after adjusting for critical covariates. 

### 3.3. Association between Pb Exposure and Cardiovascular Disease Mortality

We explored the association of cardiovascular disease mortality risk with BLL, AL, and the interaction of AL and BLL (AL X BLL) using cox proportional hazard ratios. The results indicated that cardiovascular mortality was 2.35 times higher in those with BLL above the 50th percentile in the US population as compared to below. The results also found that the combined effect of Pb and AL was 1.82 times higher in those with BLL above the 50th percentile and AL above 3 as compared to below. The results are found in Table 3 below.

## 4. Discussion

Understanding the totality of exposures that contribute to cardiovascular disease mortality is critical to mitigating the leading cause of death in the United States and world. These high rates can be lowered only if the causes are understood. This study adds to the literature on the effects of social and environmental exposures on cardiovascular disease mortality. 

With adjustment for demographic factors and some important potential confounders, we observed a positive association between blood Pb and CVD mortality and the interaction of blood Pb and chronic physiological stress with cardiovascular disease mortality.

Race/Ethnicity was a critical factor in exposure. Even though non-Hispanic Whites are the largest segment of the US population in this study, and represented a large proportion of those with BLL above the 50th percentile, they were proportinally less exposed to Pb. Specifically, there was a substantial increase in the proprortion of non-Hispanic Blacks and Mexican Americans that were above the 50th percentile in BLL compared to below it, compared to other ethnicities suggesting that these ethnicities are more likely to be exposed to Pb as compared to their proportion in the population. This matches the work of others, which found that Pb concentrations were more elevated in non-Hispanic Blacks and Mexican-Americans than Whites [54].

The results finding an association of BLL with AL suggest that although BLLs of adults have continued to decline, the health impact of Pb and physiological stress are intricately linked. This may either mean that environments which have Pb tend to be filled with more stressors or it may indicate that those who are chronically stressed are more likely to work, live in homes, or consume Pb-contaminated items. In addition, because Pb exposure and stressors tend to occur concurrently with low socioeconomic status, these findings will likely have a more significant impact among these populations [55]. These results match the work of Lindgren and colleagues who found in a study of occupationally-exposed patients that cumulative Pb exposure was associated with general distress [56].

Our findings suggest that BLL may be more critical than AL in bringing forth cardiovascular disease mortality. The reason for this is difficult to disentangle in a study of this sort and would need lab-based methods to assess the mechanistic differences between both exposures. We hypothesize that varying degrees of inflammation and oxidative stress produced by both processes are likely critical in any differences that may be produced. 

The exposure to environmental contaminants is rarely isolated. Most human beings are exposed to a complex mixture of many environmental [57,58] and social exposures [59,60], and the physiological manifestation of them determines the biological response both in the short and long term. The exposome embodies this concept and reflects the reality of how exposure occurs [61]. The co-occurrence of elevated Pb burden with higher levels of chronic stress increases the possibility of interactions of these risk factors. Further support for such a possibility derives from the fact that both Pb exposure and stress stimuli (through the hypothalamic-pituitary-adrenal [HPA] axis) act on dopamine/glutamate mesocorticolimbic systems of the brain [62,63]. 

AL and subsequent biological dysregulation bring forth cardiovascular disease [35,64,65]. When combined with Pb, the outcome can result in cardiovascular disease mortality in adult populations. 

The results of this work suggest that public health agencies must target their messaging about behavioral interventions such as diet, exercise, and other resilient behaviors along with environmental messages about Pb exposure risk in order to holistically alleviate cardiovascular disease risk. In addition, prevention of Pb exposure risk from water [66], soil [67,68], and the air [69] is critical to mitigating risk. This will require governments to monitor water infrastructure and remediate areas impacted by legacy Pb.

Finally, more must be done to educate children in school environments about the risk of Pb exposure by giving the teacher the requisite knowledge and strategies [70] about the dangers of Pb exposure to provide students with the agency to mitigate Pb exposure risk at all stages of their lives.

Limitations of this study include blood Pb levels being used for the analysis. BLL represents acute exposure to Pb as the half-life for Pb in the blood is roughly 28 days. Bone Pb levels or Zinc protoporphyrin (ZPP) analysis would have given a better indication of more long-term exposure. Measuring lead in plasma may also have improved the study, as plasma lead levels may better reflect Pb that is more freely available for exchange with target tissues than Pb levels in whole blood [71]. Also, AL may not fully capture the experience and full impact of chronic stress related to health; however, it offers critical insight and is an accepted method to use with NHANES data [23,24,25,26].

Future work should explore these findings in a longitudinal study among various age groups. Indeed, as individuals age, their Pb exposure levels increase [72] due to its biological persistence, while stress levels have been found to decrease [73] with age in some studies. Future work should seek to understand the exact point in the life course where the Pb–stress interaction produces the most risk for cardiovascular disease mortality.

## 5. Conclusions

In summary, US Adults exposed to Pb are more likely to have elevated chronic stress levels. Finally, the combined effect of Pb and chronic physiological stress has a significant influence on cardiovascular disease mortality.

## Figures and Tables

**Table 1:** Means (stderr) and prevalence of analysis variables across BLL levels above and below the 50th percentile in US population.

	BLL above 50th Percentile	BLL below 50th Percentile
Allostatic Load	2.16 (0.022)	2.10 (0.024)
Gender	M: 61.7 (0.51)	M:36.3 (0.51)
	F: 38.33 (0.51)	F: 63.7 (0.51)
BMI	27.8 (0.081)	29.5 (0.123)
Race/Ethnicity		
A) White	A) 70.1%	A) 72.2%
B) Black	B) 11.3%	B) 10.3%
C) Mexican American	C) 8.5%	C) 7.3%
D) Other Hispanic	D) 5.0%	D) 5.4%
Alcohol	0.06%(0.03)	0.02% (0.01)
Smoking	2.3% (0.20)	1.23%( 0.16)

**Table 2:** Association between AL and BLL.

**Linear Regression**	N = 4268	Unadjusted AL beta	*p*-value	* Adjusted AL beta	*p*-value
	BLL dichotomized at 50%	0.0657 (0.023)	0.005	0.255 (0.043)	0.0001
**Logistic** **Regression**	N = 4268	Unadjusted AL 3 OR	*p*-value	* Adjusted AL 3 OR	*p*-value
	BLL dichotomized at 50%	1.11 (0.05)	0.015	1.52 (0.113)	0.0001
	N = 4268	Unadjusted AL 4 OR	*p*-value	* Adjusted AL 4 OR	*p*-value
	BLL dichotomized at 50%	1.16 (0.75)	0.029	1.73 (0.168)	0.0001

* Adjusted for gender, BMI, smoking, alcohol consumption, country of birth, and income.

**Table 3:** Cardiovascular mortality risk in adults exposed to Pb and Stress.

	Unadjusted HR	*p*-Value	* Adjusted HR	*p*-Value
BLL	2.94 (0.293)	0.0001	2.35 (0.298)	0.0001
AL	0.984 (0.135)	0.908	1.078 (0.101)	0.435
AL X BLL	1.96 (0.323)	0.0001	1.82 (0.370)	0.014

* Adjusted for gender, BMI, alcohol consumption, smoking, country of birth.

## Data Availability

The NHANES dataset is publicly available online, accessible at cdc.gov/nchs/nhanes/index.htm (Accessed on 18 June 2020).
